# Ballistic Heat Transport in Nanocomposite: The Role of the Shape and Interconnection of Nanoinclusions

**DOI:** 10.3390/nano11081982

**Published:** 2021-07-31

**Authors:** Paul Desmarchelier, Alice Carré, Konstantinos Termentzidis, Anne Tanguy

**Affiliations:** 1 Univ Lyon, INSA Lyon, CNRS, CETHIL, UMR5008, 69621 Villeurbanne, France; paul.desmarchelier@insa-lyon.fr (P.D.); alice.carre@insa-lyon.fr (A.C.); 2 Univ Lyon, INSA-Lyon, LaMCoS, CNRS, UMR5259, 69621 Villeurbanne, France; 3 Univ Lyon, CNRS, INSA Lyon, CETHIL, UMR5008, 69621 Villeurbanne, France; Konstantinos.termentzidis@insa-lyon.fr; 4 ONERA, University Paris-Saclay, Chemin de la Hunière, BP 80100, 92123 Palaiseau, France

**Keywords:** nanoinclusion, ballistic transport

## Abstract

In this article, the effect on the vibrational and thermal properties of gradually interconnected nanoinclusions embedded in an amorphous silicon matrix is studied using molecular dynamics simulations. The nanoinclusion arrangement ranges from an aligned sphere array to an interconnected mesh of nanowires. Wave-packet simulations scanning different polarizations and frequencies reveal that the interconnection of the nanoinclusions at constant volume fraction induces a strong increase of the mean free path of high frequency phonons, but does not affect the energy diffusivity. The mean free path and energy diffusivity are then used to estimate the thermal conductivity, showing an enhancement of the effective thermal conductivity due to the existence of crystalline structural interconnections. This enhancement is dominated by the ballistic transport of phonons. Equilibrium molecular dynamics simulations confirm the tendency, although less markedly. This leads to the observation that coherent energy propagation with a moderate increase of the thermal conductivity is possible. These findings could be useful for energy harvesting applications, thermal management or for mechanical information processing.

## 1. Introduction

Many applications in electronics require materials with tailored mechanical, electronic or thermal properties. To this end, the appropriate element, alloy, phase, crystallinity or a combination of them can be chosen. Nanostructuration allows a further improvement of performances. A wide variety of nanocomposites exists, one of the simplest consisting of nanoinclusions (NIs) of a different phase or material embedded in a host matrix. Crystalline NIs in a crystalline matrix are used for many applications, such as thermoelectric generation [1]. For the same application, crystalline NIs in an amorphous matrix have also been proposed [2]. This last possibility takes advantage of the low thermal conductivity of the amorphous matrix while retaining some electronic transport properties of the added crystal.

However, the NIs and matrix influence each other [3,4], notably their vibrational and thermal properties. A better understanding of the interaction of the nanoinclusions and matrix is crucial to further improve the performances of these nanocomposites.

To study the heat dissipation through an amorphous/crystalline nanocomposite, one should understand both the physics of the amorphous material and that of the crystalline nanoclusters. The modern understanding of thermal transport in glasses was laid by Allen and Feldman [5,6]. They introduced an intermediary transport regime between the localization and the propagation of vibrational modes: the diffusive regime. They established a distinction between propagative and non-propagative modes. In the former, the phonon gas model can be applied, but not in the latter, due to strong scattering. However, some non-propagative modes still contribute to the thermal conductivity through energy diffusion. However, the distinction between propagative and diffusive modes is still under discussion; some authors argue that each mode has to be distinguished individually [5,7] and others use a frequency limit to discriminate between propagative and diffusive modes [8,9]. The used frequency limit is often set by the Ioffe–Regel criterion. This criterion relies on the comparison between the mean free path (MFP) and the wavelength [10]. The sometimes blurred boundary between propagative and diffusive modes has led other authors to claim that a clear distinction between the two is not meaningful [11].

The introduction of NIs in a solid matrix modifies the behavior of the material. For instance, a particle array can act as a low-pass filter, scattering the higher frequencies [4]. Different parameters have different effects: the rigidity contrast impacts the scattering and eventually pins the energy [12]. A higher surface to volume ratio is known to decrease the effective thermal conductivity [13]. Less instinctively, it has been shown in the same study that the relative crystalline orientation between the particles also modifies the thermal conductivity of the material, banning or promoting the phonon percolation. The size distribution of the NIs has also been proposed to reduce the thermal conductivity of crystal–crystal nanocomposites [14]. Finally, the presence of NIs can cause an anticipation of the transition from propagative to diffusive regime in amorphous/crystalline nanocomposites [15].

Many approaches have been proposed to model the effective properties of multiphase materials, such as amorphous crystalline nanocomposites [16]. These models generally use the bulk properties of the materials and can include some variation to take size and interface effects into consideration. However, at the nanoscale, the intrinsic properties of the materials can change, for instance with their size [17]. These variations render the predictions based on the bulk properties difficult; for instance, in the case of orientation [13], the effective medium approach proposed [18] fails and the microstructure has to be explicitly considered.

Most of the theoretical studies of NIs’ impact on the vibrational and thermal properties assume that NIs are spherical [4,12,13,15]. However, NIs can have multiple shapes [19]. The shape influences the properties, for instance, NIs with a high surface to volume ratio increase the electrical conductivity in polymers [20]. This ratio similarly increases the heat transport in nanofluids [21]. Moreover, when the mass fraction of NIs is high enough, the NIs can form a percolating network [22]. For Si NIs in a SiO_2_ matrix, the percolation can be controlled and modifies the properties of the material [23,24].

A percolating network of NIs is similar to a nanomesh embedded in an amorphous matrix. Embedded nanowire (NW) meshes are already used in polymers to increase their thermal conductivity [25]. More generally, Car et al. showed that it is possible to obtain single crystalline nanowire meshes (NW-M) [26]. These NW-M, in 2D or 3D, are also known to have a low thermal conductivity compared to bulk material [27,28]. Finally, a crystalline/amorphous nanocomposite is comparable to nanocrystalline materials. For these materials, studies exist about the transmission of phonons, across a single interface [29] or across multiple grain boundaries [30]. The grain size and grain-size distribution also impact the transport [31].

The purpose of this paper is to gain a better understanding of the effect of the gradual interconnection of crystalline NIs on thermal conductivity and ballistic transport.

To this end, several structures are studied, using equilibrium molecular dynamics (EMD) to compute their thermal conductivity and the wave packet propagation method to distinguish propagative and diffusive contributions. After the description of the configurations used, the different analysis methods are presented. First, the qualitative impact of the inclusion at different frequencies is considered, and then the vibrational properties of the different configurations are studied. These properties are used to estimate the thermal conductivity, via the kinetic theory of gases framework, and then this thermal conductivity is compared to the results obtained with the EMD methodology. Finally, the impact of ballistic transport and NIs’ interconnections on the effective thermal conductivity are discussed.

## 2. Materials and Methods

### 2.1. Studied Configurations

The nanocomposites studied here are composed of crystalline Si (c-Si) NIs embedded in an amorphous Si (a-Si) matrix. The NIs’ shapes and interconnections are varied to study their impact on the effective thermal conductivity and on the ballistic transport. The NIs are gradually interconnected, from an array of spherical NIs to a 3D nanowire mesh. The host matrix is an amorphous Si cube of side 11.9 nm, containing 84 × 10^3^ Si atoms for a density of 2.32 $g{\mathrm{cm}}^{-3}$, cut out of a larger sample obtained in a previous study [15]. This length is adapted to get an integer number of crystalline primitive cells and thus a monocrystal in case of structural percolation. Periodic boundary conditions are used in all directions.

The nanocomposites are formed in the following manner: the NI shape is first hollowed out of the matrix, and then filled by crystalline Si (c-Si). The added crystal has the 〈100〉 direction aligned with the *x* axis. In order to avoid the superposition of atoms when the crystalline phase is added, the holes are larger than the NIs themselves by 0.1 *Å*. The created NIs have the same volume, so that all configurations have the same crystalline fraction (30% of crystalline phase overall). There are 26 × 10^3^ crystalline atoms out of the 84 × 10^3^ total; the exact number of atoms varies by a few hundred in the different configurations. Four shapes of NIs are considered: a sphere (S) with a radius of 5 nm (see Table 1, second column); a sphere with six conical extremities pointing in the Cartesian coordinate directions without reaching the edges of the simulation box (see Table 1, third column) that is referred to as sphere with cones (SC); the third is similar to the former but has longer conical extremities that reaches the simulation cell boundaries (see Table 1, fourth column) and is referred to as sphere with truncated cones (STC); a 3D crossing of three nanowires of 2.5 nm in radius aligned with the Cartesian coordinates (see Table 1, last column) that is referred to as nanowire mesh (NW-M). The box size was set around this last NI shape. All NIs are centered in the host matrix. For the SC configuration, the central sphere has a radius of 4.6 nm. The added cones have an opening angle of 100${}^{\circ }$ and a height of 3.0 nm. The base of the cones (circle) correspond to the intersection of the sphere by a plane 2.9 nm away from the center of the sphere/host a-Si matrix. The apexes of the cones are 0.3 nm away from the simulation box edges and the bases of the cones are prolonged until they intersect with the central sphere. This results in a neck of 0.6 nm between two inclusions. For the STC configuration, the central sphere has a radius of 4.2. The cones have a radius of 1.0 nm at their junction with the box boundary. Their opening angle is of 67${}^{\circ }$ and total height of 3.0. Again, the base of the cones (circle) correspond to the intersection of the sphere by a plane 2.9 nm away from the center of the sphere/host a-Si matrix. Thus, only the STC and the NW-M have a continuous crystalline path across their simulation box; this continuous crystalline path across the structure is referred to as crystalline structural percolation. This structural percolation has a minimum diameter of 2 nm for the STC and of 5 nm for the NW-M. Additionally, we study a porous sample, with spherical pores of the same diameter as the S system (see Table 1 first column), and a fully amorphous sample is also studied for the sake of comparison. This porous configuration contains 58 × 10^3^ atoms. In Table 1, the different NIs are represented in 3D in the first row, and in the second row a cross-section at the middle of the corresponding nanocomposite is depicted. These representations are obtained thanks to OVITO [32].

After the geometrical construction, the different configurations are annealed in the following manner. The atomic positions are relaxed using a conjugated gradient (CG) method, then the system is annealed at 100 K for 1 ps and finally a second conjugated gradient force minimization is performed. (At this point, we should mention that the relaxation induces small reconfigurations at the surface of the NIs. This interfacial reconfiguration causes a reduction of up to 3% of the crystalline fraction and can slightly change the surface to volume ratio of the NIs. To take this into account, when computing the surface to volume ratio, only the particles recognized as diamond structure and first and second neighbors by a modified common neighbor analysis [33] are considered.) All modeling and MD simulations are carried out using the open-source software LAMMPS [34].

We used a modified Stillinger–Weber potential [35] for its more realistic modeling of the interfaces between c-Si and a-Si in terms of interfacial energy and of atomic energies inside the two phases [36].

### 2.2. Equilibrium Molecular Dynamics

The equilibrium molecular dynamics (EMD) method is used to estimate the thermal conductivity (κ) of the configurations previously described. This method relies on the fluctuation dissipation theorem linking the decay of the fluctuation of an internal variable to its response function. Here, the flux auto-correlation integral is linked to the thermal conductivity using the Green–Kubo formula [37]:

(1) $$\kappa_{\alpha \beta }={\left(Vk_{B}T^{2}\right)}^{-1}\int_{0}^{\infty }\langle J_{\alpha }\left(0\right)J_{\beta }\left(t\right)\rangle dt$$

with α and β the directions, *V* the volume of the system, $k_{b}$ the Boltzmann constant and $J_{\beta }\left(t\right)$ the thermal flux in the direction β computed at a time *t*. The thermal flux is computed by LAMMPS using atomic energy for the convective part and the “group” atomic stresses for the virial contribution [38]. A discretized version is [39]:

(2) $$\kappa_{\alpha \beta }=∆t{\left(Vk_{B}T^{2}\right)}^{-1}\sum_{m=1}^{M}{\left(m-p\right)}^{-1}\sum_{n=1}^{p}J_{\alpha }\left(m+n\right)J_{\beta }\left(m\right)$$

with ∆t the time step between two successive flux computations, *M* the total number of time steps and *p* the number of time step over which the auto-correlation function is averaged.

Before computing the flux auto-correlation function, the configurations (one inclusion in the amorphous cube as represented in the bottom row of Table 1) are first heated at 50 K, using a random initial velocity distribution. After that, the temperature is increased from 50 to 600 K at constant pressure in 0.05 ns, that is 1 × 10^5^ time steps. Then, the system is annealed at 600 K with a Nosé–Hoover thermostat for 0.25 ns (5 × 10^6^ time steps) to ensure better temporal stability. To insure the absence of recrystallization, it is checked that this annealing does not impact the radial distribution function at 300 K. After this annealing, the temperature is decreased to 300 K at constant pressure in 0.05 ps and then equilibrated at 300 K for 2 ns (4 × 10^7^ time steps). The flux auto-correlation function is finally measured during 10 ns (2 × 10^8^ time steps) in a constant energy simulation, using a velocity Verlet integration scheme. For all simulations, a time step of 5 × 10^−7^ ns is used. For the computation of the auto-correlation, the flux is sampled every 1 × 10^−5^ ns and the flux auto-correlation decay is computed over 0.04 ns. These simulations are repeated 5 times, with a different initial velocity distribution for each repetition, to get better statistics. The final value is the mean κ across the simulations and the uncertainty range is defined by the highest and lowest values of the individual runs.

### 2.3. Thermal Conductivity from the Kinetic Theory

The thermal conductivity of the different configurations can also be evaluated using their vibrational properties; for this, the method initially developed by Tlili et al. [15] for spherical NIs is used. The contribution of the propagative and the diffusive modes are separated. The propagative contribution ($\kappa_{P}$) is estimated with the following integral [40]:

(3) $$\kappa_{P}=\sum_{\eta }\int_{0}^{\nu_{max}}m_{\eta }C\left(\nu ,T\right)v_{\eta }^{2}\left(\nu \right)\tau_{\eta }\left(\nu \right)g_{\eta }\left(\nu \right)d\nu$$

with C(ν,T) the heat capacity at the temperature *T* and frequency ν, $v_{\eta }\left(\nu \right)$ the group velocity, $\tau_{\eta }\left(\nu \right)$ the phonon lifetime, $g_{\eta }\left(\nu \right)$ the density of state at the frequency ν and $m_{\eta }$ the degree of freedom associated to the polarization η (longitudinal or transverse). $\nu_{max}$ is the frequency for which the group velocity is zero or ill-defined.

The contribution of the diffusive part ($\kappa_{D}$) can be estimated through [41]:

(4) $$\kappa_{D}=\int_{0}^{\nu_{maxD}}3C\left(\nu ,T\right)g\left(\nu \right)D\left(\nu \right)d\nu$$

with D(ν) the diffusivity at the frequency ν. $\nu_{maxD}$ is the frequency at which the diffusivity is considered negligible, that is 15 THz for the configurations studied. The heat capacity, using the Debye model [40], is given as follows:

(5) $$C\left(\nu ,T\right)=\frac{Nk_{b}}{V}{\left(\frac{2\pi \nu \hbar }{k_{b}T}\right)}^{2}\frac{\exp\left(\frac{2\pi \nu \hbar }{k_{b}T}\right)}{{\left(\exp\left(\frac{2\pi \nu \hbar }{k_{b}T}\right)-1\right)}^{2}}$$

with $k_{b}$ the Boltzmann constant, *ℏ* the Planck constant, *V* the volume and N the number of atoms. The methods for the estimation of other components of Equations (3) and (4) are detailed in Section 2.4.

The global thermal conductivity is taken as the sum of the diffusive and propagative contributions. Here, as both propagative and diffusive behaviors appear at most frequencies, both contributions are considered over the whole spectrum.

### 2.4. Wave Packet Propagation

The wave packet (WP) method is used to study the different aspects of the phononic contribution to the thermal conductivity [42]. This method enables the estimation of the MFP and diffusivity in a dual wave/particle description of phonons. These quantities are estimated thanks to the excitation of different vibrational modes and the measure of their decay rate according to space and time.

First, the media in which the WP propagates is obtained from the repetition of the cubes described in Section 2.1. They are repeated 6 times in the *x* direction. Indeed, a sufficiently long sample is needed to study the spatial decay of the WP. Before the excitation, the atomic velocities are set to 0 and the position of the atoms are relaxed using a CG method to minimize the force so that any movement of the atoms is caused by the WP. Then, an excitation is applied in a central slice of 0.2 nm between two repetitions of the initial configuration. This excitation is a Gaussian windowed sinusoidal force impulsion,

(6) $$f=A\sin\left[2\pi \nu \left(t-3\tau \right)\right]\exp\left[-\frac{{\left(t-3\tau \right)}^{2}}{\left(2\tau^{2}\right)}\right].$$

The amplitude *A* is chosen to be sufficiently low to avoid anharmonic effects (here 3.773 × 10^−4^ eV/Å). The Gaussian window width τ balances between spatial extension of the WP compared to the nanocomposite length, and the resolution in the frequency space, here 36 × 10^−4^ ns. The studied frequencies range from 1 THz, which is the limit of the resolution due to the τ used, to 15 THz by increments of 1 THz.

The force *f* can be applied parallel to the principal dimension, creating longitudinal (L) waves or perpendicular to it creating transverse (T) waves. Alternatively, the force can be applied in a random direction, different for each atom, preventing the formation of a coherent wave. This random excitation with a uniform angle distribution is used to compute the energy diffusivity [8].round-precision = 2 After the impulsion, the kinetic energy as a function of position over the *x* axis is recorded every 1 × 10^−5^ ns from the creation of the impulsion until the wave fronts reach the periodic boundaries.round-precision = 1 The resolution along *x* is of 0.72 nm. Additionally, the position and kinetic energy of every atom are recorded every 3 × 10^−4^ ns, in order to get a spatially resolved energy distribution. For these simulations, a velocity Verlet integration scheme was used with a time step of 1 × 10^−6^ ns.

**Figure 1:**
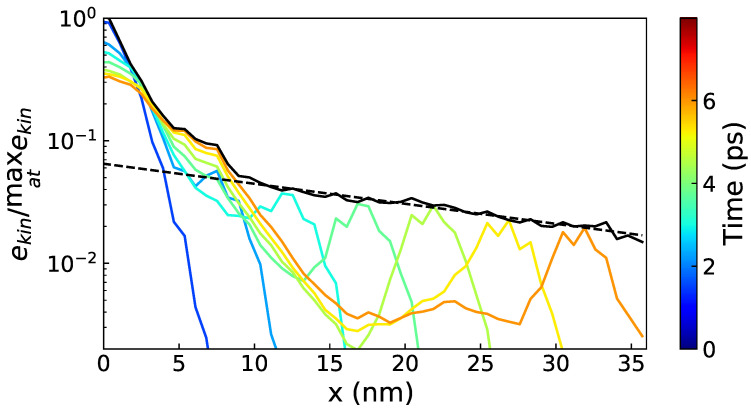
Representation of the propagation of an 8 THz longitudinal impulsion in the NW-M, with the energy distribution at different time steps (colored lines), the envelope defined as the maximal value of the kinetic energy at a given position (black solid line) and the exponential fit used to compute the MFP (dashed black line).

The MFPs of the propagating modes are estimated from the decay rate of the envelope of the WP as a function of the distance to the excited slice. The envelope of the WP is defined as the maximum value of the kinetic energy at each point along the propagation path (see Figure 1). The envelope of a WP traveling ballistically follows a Beer–Lambert law (exponential decrease) [42]. Due to the presence of the NIs, the envelope may contain plateaus and sharp decreases; thus, to get a meaningful value of the exponential decay fit, the portion on which the least square fit is made has to be chosen appropriately. Moreover, as shown in Figure 1, in the vicinity of the excited slice a diffusive part is visible; this part is not included in the MFP computation. The propagation takes place in both *x* positive and *x* negative directions, and the final value of the decay rate is the average of the two.

At high frequencies, the exponential decay can be ill-defined. This is the case for configurations without NIs above 12 THz for the longitudinal polarization and for frequencies above 7 THz for the transverse polarization. In those cases, the decay does not follow an exponential attenuation. The penetration length is then used instead of the MFP. The penetration length is defined as the distance to the excitation point for which the energy as been divided by *e* [43]. This corresponds to the MFP in the case of a perfect exponential attenuation.

The energy diffusivity is estimated with the method described by Beltukov et al. [8]. This is done after a random force excitation, to cancel the propagative (coherent) part. The average square distance to the diffusion front for each frequency is computed as:

(7) $$R^{2}\left(t\right)=\frac{1}{E_{tot}}\sum_{i=0}^{N}x_{i}^{2}E_{i},$$

with *N* the number of slices, *i* the slice index, $x_{i}$ the distance to the excitation and $E_{i}$ the kinetic energy of the ith slice. The diffusivity is linked to the time dependence of $R^{2}$ by the equation of one-dimensional diffusion,

(8) $$R^{2}\left(t\right)=2D\left(\nu \right)t.$$

In each case, D(ν) is computed through a least square fit of $R^{2}\left(t\right)$.

### 2.5. Lifetime Estimation and Temperature Effect

The computation of the thermal conductivity through Equation (3) relies on the estimation of the phonon lifetime as a function of frequency and polarization. The lifetime is considered to be limited by two phenomena: interfaces or defect scattering and the phonon–phonon scattering. The former is assumed to be geometry dependent only and is estimated thanks to the MFP and the group velocity:

(9) $$\tau_{geom}^{-1}=\frac{v_{\eta }\left(\nu \right)}{\Lambda_{\eta }\left(\nu \right)}$$

with $\Lambda_{\eta }\left(\nu \right)$ the MFP at frequency ν.

When the wave-packet propagation simulation takes place at 0 K, the reduction of lifetime due to anharmonicity is underestimated. To compensate for this, a lifetime due to phonon–phonon interactions is introduced. This lifetime is estimated with the empirical relation described in the Callaway model as a function of temperature and frequency [44].

(10) $$\tau_{ph-ph}^{-1}=P{\left(2\pi \nu \right)}^{2}T\exp\left(-C_{U}/T\right)$$

with *P* and $C_{U}$ empirical scattering parameters; the used values are those of crystalline bulk silicon found in the work of Yang et al. [45].

The global lifetime used in Equation (3) is then estimated using Matthiesen summation rule:

(11) $$\tau^{-1}=\tau_{geom}^{-1}+\tau_{ph-ph}^{-1}.$$

### 2.6. Group Velocity through the Dynamical Structure Factor

The dynamical structure factor (DSF) is a spatial and temporal Fourier transform of the atomic displacements used to characterize the vibrational properties of a system. This is very similar to what can be measured by X-ray or neutrons scattering experiments [46]. It is defined as:

(12) $$S\left(q,\omega \right)=\frac{2}{NT}{\left|\sum_{i}^{N_{at}}\exp\left(-iq\;\cdot \;r_{i}\right)\int_{0}^{\tau }u_{i}\left(r_{i},t\right)m_{\eta }exp\left(i\omega t\right)dt\right|}^{2}$$

with q the wave vector, $u_{i}$ and $r_{i}$ the displacement and position of the ith atom, $m_{\eta }$ the polarization vector (parallel or perpendicular to q), *T* the temperature and *N* the total number of atoms [4].

The resolution of the wave vector is given by 2π/L with *L* the length of the simulation box in the direction of the wave vector. The direction of the vector q can be chosen arbitrarily to match the different direction in the reciprocal lattice space (here that of c-Si).

**Figure 2:**
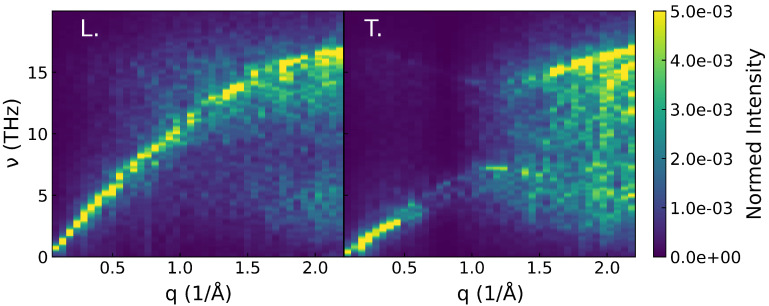
Dynamical structure factor computed through Equation (12) for the NW-M in the ΓX direction for the longitudinal (**left**) and transverse polarization (**right**).

The atomic trajectories used for the computation of S(q,ω) are obtained in the following manner: the sample is heated at 100 K and equilibrated at this temperature for 5 × 10^−3^ ns using a Nosé–Hoover thermostat. After this, the atomic trajectories are recorded during a 1 × 10^−2^ ns long constant energy simulation. An example of DSF is displayed in Figure 2.

From the DSF, the phononic dispersion curves can be obtained. First, the DSF is filtered through a convolution with a typical energy resolution curve of line-width 1.35 meV (as suggested by Tlili et al. [15]). Then, for a given wave-vector direction, the dispersion is estimated from the frequency for which S(q,ω) has the highest value for each wave vector within the acoustic phonons frequency range. This dispersion is finally fitted to a sine function allowing the analytical derivation of the group velocity as a function of frequency. The expression of the group velocity contains an arcsin function; thus, when the frequency is outside of the definition domain, it becomes ill-defined and is considered nil. To get the appropriate dispersion, q is chosen as the propagation direction of the WP. This corresponds to the 〈100〉 crystalline orientation in the direct space or to ΓX in the reciprocal space. An alternative method to estimate both the dispersion relation and the lifetime from the DSF is discussed in Appendix A.

### 2.7. Vibrational Density of States

The vibrational density of states (VDOS) of the different configurations is evaluated with the Fourier transform of the velocity auto-correlation function (VACF) [47]. Before computing the VACF, the system is equilibrated at 50 K for 0.1 ns with a Nosé–Hoover thermostat. The VACF averaged over all the atoms is then recorded over the next 0.1 ns without thermostat. To obtain the final VDOS, the Fourier transform of the VACF is filtered using a Savitzky–Golay polynomial filter [48].

Additionally, the VDOS of the amorphous Si was computed using the dynamical matrix [47] on a smaller sample. The square roots of the eigenvalues of this matrix give the eigenfrequency of the system. By distinguishing the modes that keep the volume of the Voronoi cell around each atom and those that do not, the transverse and longitudinal modes can be distinguished [49]. The VDOS is then approximated by series of Chebychev polynomials [50]. The dynamical matrix was computed for a cubic cell of side 4 nm with periodic boundary conditions containing 3159 atoms and the Voronoi cells determined thanks to the *Voro++* open-source software [51].

## 3. Results

### 3.1. Ballisticity through Wave-Packet Simulations

A qualitative analysis of the time evolution of the kinetic energy distribution can give physical insights into the impact of nanostructuration on energy propagation. Table 2 shows the atomic kinetic energy on a cross-section for the different configurations after a 2 THz impulsion. The impulsion is made in the middle of the system and propagates in both the negative and positive *x* directions. The two directions being symmetric, only one direction (*x* positive) is represented. The first half of the table corresponds to longitudinal polarization. The main observation for most configurations is that most of the energy travels through the sample as a plane wave. The NIs do not strongly affect the propagation at this frequency: the WP travels through the nanocomposites and the a-Si similarly. However, there is still some scattering visible through the small spots of high energy concentration after the passage of the WP. These spots are mainly located in a-Si and at the interfaces between the NIs and matrix. For the porous configuration, a plane wave is also visible, although its intensity is strongly reduced by the time it reaches the end of the simulation box. However, more importantly, most of the energy stays in the center and slowly diffuses through the sample.

For the transverse waves at the same frequency (displayed in the second half of Table 2), the dispersion is more marked. The vertical red lines, characteristic of plane waves, can be distinguished in the first few images, but disappear before reaching the simulation box boundary. The waves are quickly scattered, even for bulk a-Si. In the configurations containing NIs, the vertical lines materializing the plane waves are distorted. This distortion of the wave-front is due to the WP traveling more quickly in the crystal than in the glass matrix. The porous configuration is again the configuration for which the scattering is the strongest.

To summarize the low frequency WP propagation, one can observe that the shape of the NIs has no impact on either the longitudinal or transverse waves. The longitudinal plane-waves preserve their shape for both interconnected and not interconnected NIs, and the transverse waves are diffused quickly. The situation is quite different for the nanoporous amorphous silicon, for which the plane waves disappear rapidly for both polarizations. We stress the fact that an amorphous/crystalline nanocomposite could be “transparent” to low frequency longitudinal waves.

The behavior of the nanocomposites after a high frequency impulsion is displayed in Table 3. The first part contains the evolution of a longitudinal WP at 10 THz or two third of the maximum frequency for which a group velocity can be defined. It appears that there is no propagation in the amorphous matrix. For all configurations, the energy slowly spreads through the amorphous matrix.

However, on top of this diffusion, a propagative behavior limited to the crystal also appears. This is particularly noticeable in the case of structural percolation. In this case, the wave packet takes an oval shape and travels through the structural percolation. In the absence of percolation, the propagative part of the WP is scattered at the first crystalline/amorphous interface.

**Figure 3:**
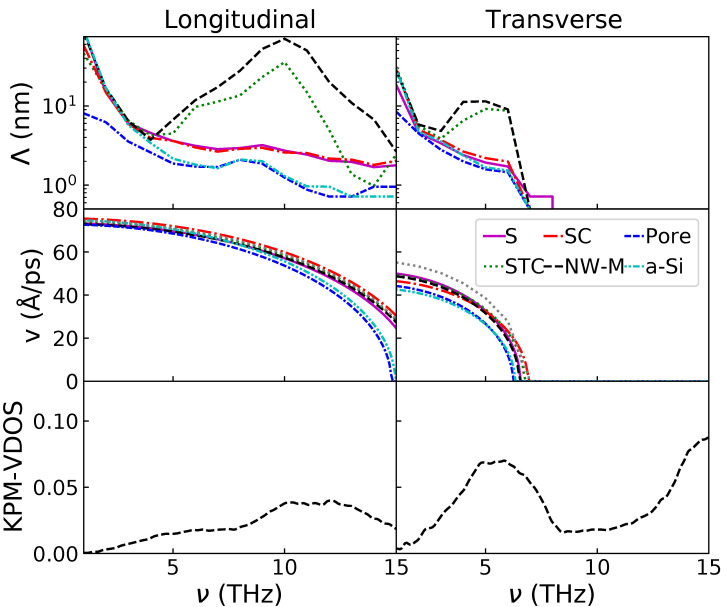
From right to left: First row, longitudinal and transverse mean free path; second row, longitudinal and transverse group velocity for the studied configurations; third row, longitudinal and transverse VDOS. Additionally, the group velocity computed for a fully crystalline sample is displayed with a dotted gray line.

For the transverse polarization, the selected frequency is 4 THz. As for the longitudinal polarization, this frequency corresponds approximately to two third of the frequency for which the group velocity becomes ill-defined (see Figure 3). The behavior is very similar to the longitudinal polarization: there is ballistic transport limited to the structural percolation region and a diffusive transport acting on a slower timescale. This diffusive behavior is visible close to the border, where the impulsion is made. However, in this case, both the crystalline NIs and the amorphous matrix participate in diffusive energy transport.

To summarize the WP propagation at high frequencies, there is a clear differentiation of the crystalline and amorphous phases. There is no propagation in the amorphous phase. Ballistic propagation through the sample is only possible through the structural percolation. We also observe that there is no backscattering or important deviation of energy in the perpendicular branches of the inclusions.

### 3.2. Diffusive and Propagative Contributions to the Thermal Conductivity

As described in Section 2, information extracted from the WP simulations can be used to estimate the thermal conductivity. First, the different components of the propagative contribution to the thermal conductivity ($\kappa_{P}$) are displayed in Figure 3. In the top left panel, the MFPs of the longitudinal WP for the different configurations are displayed as a function of the frequency. The MFP is estimated through the decay rate of the envelope, except at frequencies above 12 THz for the amorphous and porous configurations where the penetration length is used (see Section 2.4). These curves confirm what is visible in Table 2: the MFP is high at low frequencies for all configurations. Below 5 THz, the MFPs of the non-porous configuration are very similar. Only the pores decrease the MFPs at low frequencies. At higher frequencies, the configurations without structural percolation have a low MFP. This contrasts with the configurations with structural percolation, for which the MFP rises between 5 and 10 THz and decreases strongly after that. The MFP for those configurations, around its maximum between 8 and 12 THz, is almost one order of magnitude higher than without percolation. Moreover, the interconnection degree has an influence. The MFP is higher for the NW-M than for the STC. It is also noticeable that the porous and fully amorphous configurations have a small MFP peak around 8 THz; this peak was already observed for a-Si by Beltukov et al. [43]. It has been associated with the decreased number of transverse modes available for coupling at this frequency.

For the transverse polarization, in the top right panel of Figure 3, the behavior is similar. Above 7 THz, the MFP is substituted by the penetration length for all the configurations in order to avoid artifacts caused by a strong scattering. Below this frequency, the decay rate of the envelope is used (see Section 2.4). As for the longitudinal polarization, the MFPs of the configurations with structural percolation have a maximum. In this case, the maximum is within 5–6 THz. Without structural percolation, the MFP decreases as the frequency increases. Again, similar to the longitudinal polarization, most configurations share a very similar MFP at 1 THz, the only exception being the porous configuration, which has a lower MFP.

The group velocities for the longitudinal and transverse polarizations are displayed in the second row of Figure 3. All configurations share a very similar group velocity. This is especially true for the longitudinal polarization at low frequencies (below 5 THz). At higher frequencies, the group velocities for the amorphous and porous configurations are lower than the group velocities of the others. The *v* values of the configurations containing NIs are very similar to those of c-Si. For the transverse polarization, there is also a group velocity difference, although spanning over the whole spectrum. For this polarization, the *v* of the nanocomposites containing NIs is in between those of c-Si and a-Si. Finally, the transverse polarization has a nil velocity for frequencies higher than 7 THz.

The third row from the top contains the VDOS attributed to the longitudinal and transverse polarizations for the computation of $\kappa_{P}$. For this application, the transverse and longitudinal VDOS are computed via the kernel polynomial method (KPM) [49] on an a-Si sample. This allows for a good approximation in the 0–12 THz frequency range (see Appendix A for more detail). On these graphs, it can be noted that the maximum of VDOS at 10 THz for the longitudinal polarization and at 5 THz for the transverse also correspond to MFP maxima. Due to the higher lifetime conjunct with a high VDOS, these modes will contribute significantly to $\kappa_{P}$.

**Figure 4:**
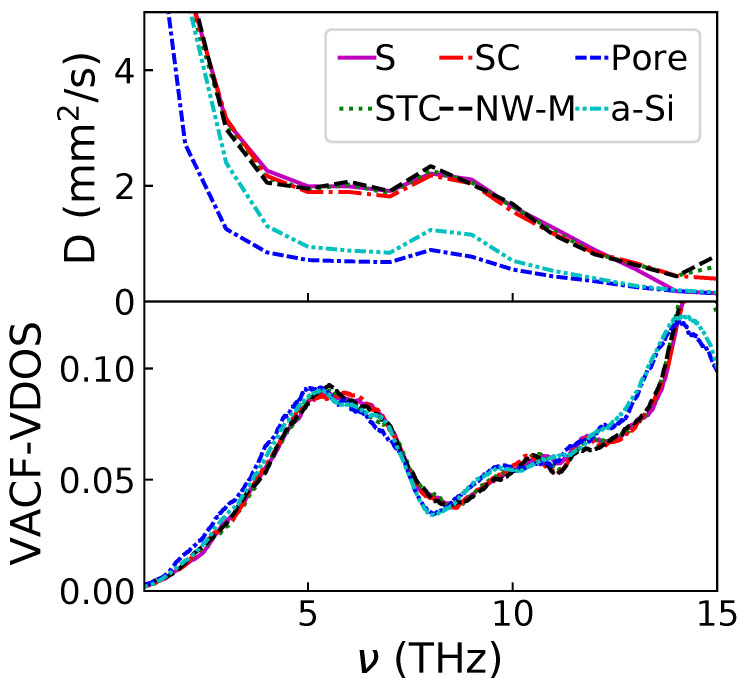
Diffusivity and VDOS as a function of frequency for the studied configurations.

The different terms contributing to $\kappa_{D}$ are shown in Figure 4. The top panel corresponds to the diffusivity computed with Equation (8). Two main observations can be made: firstly, all the configurations containing a crystalline phase share a very similar diffusivity across the whole spectrum; secondly, only the porous configuration induces a reduction of diffusivity with respect to the amorphous sample. The addition of NIs increases the diffusivity. Additionally, a small peak at 8 THz is visible for all cases; this peak corresponds to the end of the transverse phonon dispersion curve and was already observed by Allen and Feldman [6]. The VDOS computed through the VACF for the different configurations are displayed in the bottom panel of Figure 4. All the VDOS are very similar up to 14 THz. At higher frequencies, the configurations containing NIs and the others show differences. The VDOS of a-Si starts to decrease from 14 THz, while the others continue to increase. However, this difference has little effect on the $\kappa_{D}$ given that the diffusivity is very low at those frequencies.

**Figure 5:**
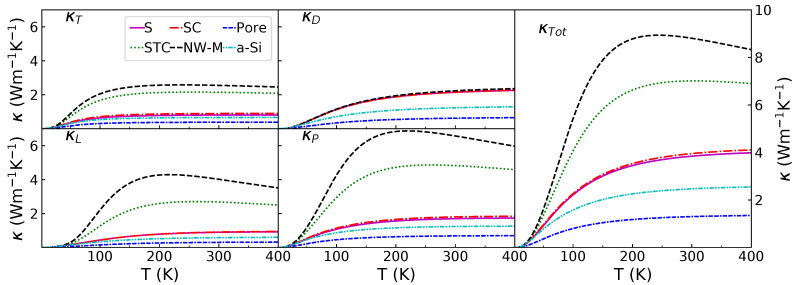
Different contributions to the thermal conductivity: the first column contains the contributions of the longitudinal phonons $\kappa_{L}$ (**bottom**) and the transverse phonons $\kappa_{T}$ (**top**); the second column the diffusive contribution $\kappa_{D}$ (**top**) and the overall propagative contribution $\kappa_{P}=\kappa_{L}+\kappa_{T}$ (**bottom**); the last columns contains the sum of the different contribution $\kappa_{Tot}=\kappa_{P}+\kappa_{D}$.

The different terms displayed in Figure 3 and Figure 4 are used to compute $\kappa_{P}$ and $\kappa_{D}$. The results for temperatures between 10 and 400 K are displayed in Figure 5. The first column contains the transverse and longitudinal propagative contribution. It confirms that the structural percolation induces a marked increase of the propagative contribution; the STC and NW-M have a larger $\kappa_{T}$ and $\kappa_{L}$. However, for the diffusive contribution in the top row of the central column, no distinction between the configurations containing NIs can be made. Only the pores seem to decrease the diffusive contribution below amorphous values. The propagative contribution, for both polarizations, increases with the degree of interconnection. When looking at the propagative contribution as a function of the temperature, it appears that $\kappa_{L}$ increases at higher temperature than $\kappa_{T}$. This is linked to the MFP peak at 10 THz and to C(T,ω) that limits the impact of high frequencies at low temperature. This important high frequency contribution also results in a maximum of $\kappa_{L}$ around 200 K for the NW-M. This is due to the empirically added phonon–phonon term (Equation (10)) that reduces the contribution of high frequency phonons as the temperature rises. The different contributions (propagative and diffusive) can be compared in the central panel. The diffusive and propagative contributions for the non-percolating configurations have similar values at 300 K.

The sum of the different contributions, $\kappa_{Tot}$, is displayed in the last column of Figure 5. At all temperatures, the same order of $\kappa_{Tot}$ is preserved. This order is, from the highest to the lowest thermal conductivity: NW-M, STC, SC and S with very similar values, then amorphous and finally the porous configuration. The maximum observed for $\kappa_{L}$ of the NW-M is still visible on the sum and happens at 244 K. Such a maxima in κ has already been predicted for SiC NWs using a similar method [52] but contrasts with experimental results on Si NW [53].

**Figure 6:**
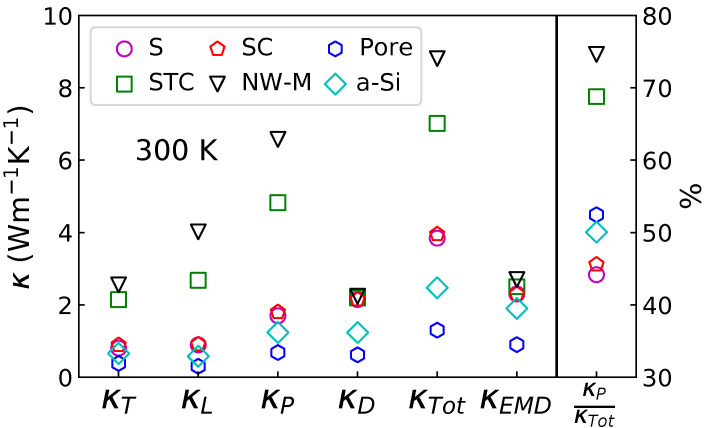
Thermal conductivities at 300 K, decomposed through Equations (3) and (4) and computed with EMD. The proportional contribution to the thermal conductivity of the propagative mode is also represented in the right part of the figure (right axis).

The different contributions to the thermal conductivity at 300 K are also shown in Figure 6. With this representation, it appears clearly that the structural percolation increases $\kappa_{P}$ and does not affect $\kappa_{D}$. As a result, the propagative part represents up to 75% of κ for those nanocomposites. This graph also shows that the addition of non-percolating NIs in an amorphous matrix increases the diffusive transport more than the propagative transport. For the S and SC configurations, the diffusive transport is dominant. Finally, it appears that, despite the overestimation of the thermal conductivity of nanocomposite containing NIs by the kinetic method compared to the results of EMD, the hierarchy in the different structures is preserved.

To briefly summarize the results obtained with the kinetic theory, it appears that as predicted previously the addition of NIs increases κ above bulk a-Si values [15]. This is due to the fact that the NIs are crystalline. This is particularly visible in the case of structural percolation, where the MFP peak at high frequencies is concomitant to a VDOS peak, resulting in a large increase of $\kappa_{P}$. This increase occurs mainly at high temperatures (see Figure 5) due to the temperature dependent frequency weighting of C(ν,T) (see Equation (5)). As high frequencies at high temperatures are also more impacted by the phonon–phonon term (Equation (10)), a maximum of $\kappa_{Tot}\left(T\right)$ appears for the NW-M. This maximum contrasts with experimental results for single nanowires of diameter similar to the NW constituting the NW-M. For these single NWs, no maximum of the thermal conductivity has been observed as a function of temperature [53]. This is a first sign that the propagative contribution, the only one which can cause the apparition of a maximum of κ, is overestimated by our implementation of kinetic theory. The evolution of κ with temperature is worth commenting: between 10 and 100 K, all configurations seem to follow the unusual $T^{2}$ power law as was observed experimentally below 1 K [54], but it can certainly not be attributed here to double well potential effect since anharmonicity is not taken into account in our simulations in this temperature range. Moreover, $\kappa_{P}$ dominates at these temperatures, contrasting with the predictions of Cahill et al. [55].

### 3.3. Global Estimation of the Thermal Conductivity

**Figure 7:**
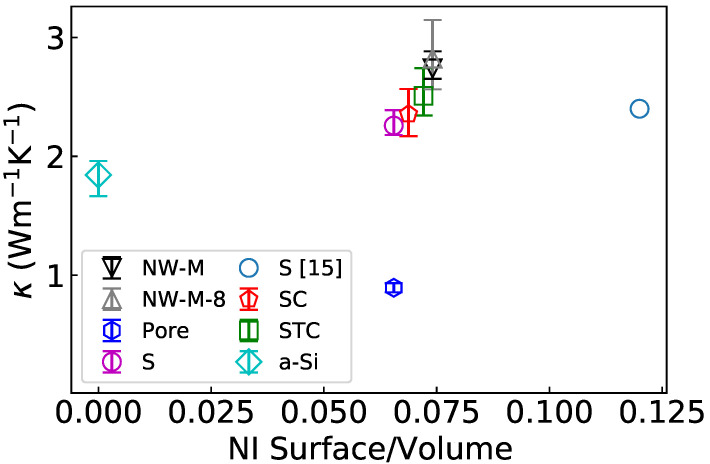
Thermal conductivity estimated with EMD for the studied configurations, as a function of their surface to volume ratio. S [15] refers to the results of Tlili et al. with the same crystalline volume fraction and NW-M-8 refers to a cube constituted by a 2 × 2 × 2 grid of the configuration NW-M.

The thermal conductivity can also be estimated from the Green–Kubo relation (Equation (2)). The results at 300 K as a function of the surface to volume ratio of the NIs are displayed in Figure 7. A clear trend appears for the configurations containing NIs; the thermal conductivity increases with the surface to volume ratio. Moreover, the thermal conductivity is very close to the one of Tlili et al. [15] for a nanocomposite with smaller NIs representing the same volume fraction but twice the surface to volume ratio. This hints that the effect of the interconnection/structural percolation is stronger than the effect of an increased scattering surface. It is also noticeable that all the NIs of this study are regrouped in the center of the graph with ratios between 6.5 × 10^−2^ and 7.5 × 10^−2^. The κ are also very close, with intersecting error bars. In the end, only the NW-M really stands out with a κ increased by 20% compared to the spherical NI. Finally, the thermal conductivity for a cubic supercell of eight NW-M (in gray) is very close to the κ of the single NW-M. This absence of variation of the thermal conductivity shows that κ does not depend on the number of NI simulated. The EMD methodology is not strongly size dependent [56,57].

The κ computed for bulk a-Si through EMD is 1.9 $W\;m^{-1}K^{-1}$, which is close to the previously reported values [9,58]. The nanoporous a-Si has a sub-amorphous κ due to the additional scattering at the surface of the pores. When the pores are filled with crystalline NIs, the κ is increased by a factor of 2.5–3 compared to the porous κ and a factor of 1.2–1.5 compared to a-Si.

The results of the EMD computations are compared to $\kappa_{Tot}$ obtained in the previous section in Table 4. As visible in Figure 6, even if the thermal conductivity predicted by Equations (3) and (4) is higher, both methods predict the same hierarchy of κ. The difference of prediction between the two methods is more pronounced for the configurations containing NIs and even more if there is a structural percolation. The last row of the table shows the results if $\tau_{phonon-phonon}$ is not taken into account. It appears that the reduction of thermal conductivity induced by this term is marked only for the configurations with a crystalline continuity.

To conclude on the EMD computation, all configurations containing NIs have a κ between 2.3 and 2.7 $W\;m^{-1}K^{-1}$ (see Table 4). The NW-M is the only configuration that has a distinctively higher κ than the nanocomposites without structural percolation. Its thermal conductivity is 20% higher than the one of the nanocomposite with a spherical inclusion. Nakamura et al. obtained experimentally a thermal conductivity between 1.7 and 1.9 $W\;m^{-1}K^{-1}$ for Si nanocrystallite of similar size in a-SiO_2_ [23]. The difference probably comes from the a-SiO_2_ having a lower κ than a-Si. In our simulation, the a-Si can be considered as a proxy for a-SiO_2_, which is a reasonable approximation if the electronic contribution is neglected as in classical MD. Moreover, counter-intuitively, when going from the spherical NI to the NW-M, κ seems to increases with the surface to volume ratio. However, usually, an increased density of interfaces leads to a reduction of κ [59]. The increased surface to volume ratio is here a consequence of the gradual interconnection of the NIs: as the shape shifts from a sphere to a NW-M, the surface to volume ratio indeed increases. In our case, the interconnection probably has a stronger effect on κ than the surface to volume ratio. In addition, a similar thermal conductivity has been found before for smaller particles having a higher surface to volume ratio but sharing the same volume fraction as the S configuration [15]. This means that the surface to volume ratio has little effect on c-Si NIs in a-Si matrix. This lack of impact of the surface to volume ratio contrasts with the results obtained for GaN NIs in SiO_2_ [60]. The origin of this difference may be found in the impedance mismatch between GaN and SiO_2_.

Furthermore, the κ of a-Si estimated here is coherent with previous results obtained with a similar method [58] as well as experimental results [61]. Porous a-Si is less studied, but the results can be compared with results obtained on porous c-Si amorphized by irradiation [62,63]. The experimental results range between 3 and 1.8 $W\;m^{-1}K^{-1}$, thus higher than the 0.89 $W\;m^{-1}K^{-1}$ obtained here. This difference can have multiple origins, two of which are important: the shape of the pores and the presence of gas in the pores in the experimental set up.

The simple effective medium approach that considers the κ of both phases, their proportion and shape overestimate the thermal conductivity [64]. Other models considering interfacial effect also fail; they predict a decrease of thermal conductivity when the surface to volume ratio increases contrary to what is visible in Figure 7 [18]. A more complex model, such as the one presented by Wang et al. [16], might be able to predict the thermal conductivity. However, as the authors pointed out, the effective model approach often fails to predict the properties at the nanoscale, where the continuum approach shows its limits.

## 4. Discussion

Previous analysis of the impact of NIs in amorphous matrices on the vibrational and thermal properties of nanocomposites via MD have focused on the intrinsic properties of spherical NIs and on their role as scatterers [12,13,15]. The influence of their shape and eventual interconnection are rarely the center of attention; here, we try to understand their role on the effective thermal conductivity and on the ballistic transport.

### 4.1. Thermal Conductivity

The gradual interconnection/structural percolation between the NIs increases the effective thermal conductivity of the studied nanocomposites. This enhancement is due to an increase of the propagative part. The diffusive part ($\kappa_{D}$) on the contrary is not affected by the shape of the inclusions or by the structural percolation. It can however be noted that $\kappa_{D}$ is increased by the introduction of NIs, and that the only way to decrease it below amorphous values is to introduce pores. Finally, we showed that the two methods used to evaluate κ conserve the same hierarchy.

Having estimated thermal conductivity through two methods (the WP method and the EMD computation), the respective results can be compared. Firstly, it appears that the two methods give slightly different results. Equations (3) and (4) of the WP method overestimate the thermal conductivity of all configurations, particularly in the case of structural percolation that makes the thermal response very inhomogeneous. It also does not take properly into account the possible thermal sensitivity of the MFP. Secondly, in opposite, the EMD simulations might not capture all the effects induced by the NIs. Additional non-equilibrium molecular dynamics (NEMD) [57] simulations containing multiple NIs could be interesting to perform. In the case of structural percolation, the heat flux will likely concentrate in the crystalline percolation and the effect of this concentration maybe lost in the flux auto-correlation over the whole sample that is used to compute κ with the EMD method.

However, the discrepancies between the values of both models also question the quantitative accuracy of the computation of κ with the kinetic theory. The robustness of the method, in particular for nanocomposites, is not established. To carry out the computations, multiple assumptions are made. These different assumptions is reviewed in Section 4.3.

### 4.2. Ballistic and Diffusive Transport

Concerning ballistic transport, the behavior at high and low frequencies must be distinguished. At low frequencies (below 5 THz), for the longitudinal polarization, no distinction can be made between the different nanocomposites containing NIs. The WP travels through NIs and matrix alike. At higher frequencies, the waves are strongly attenuated in the amorphous matrix and ballistic transport is possible through the structural percolation only.

At low frequencies, ballistic propagation was expected in the amorphous matrix [43]. Moreover, at these frequencies, there is no impedance mismatch: the group velocity in a-Si and c-Si are similar (as can be seen when comparing the $v_{L}\left(\omega \right)$ between a-Si and c-Si in the middle panel of Figure 3). The long MFP at low frequencies for a-Si/c-Si nanocomposite is consistent with results obtained with finite elements simulations [12]. Moreover, the transmission rate through a a-Si/c-Si interface is known to be high for a single interface and for grain boundaries in nanocrystalline Si [65,66]. The combination of a high MFP in the matrix, a lack of impedance mismatch and a good transmission through the interface results in a reduced impact of the NIs on the MFP at low frequencies for longitudinal polarization. For the transverse polarization, still at low frequencies (below 4 THz), the MFP is similar for all configurations, except for the porous one. This similarity happens despite the acoustic mismatch between the matrix and NIs (see Figure 3) and the stronger scattering observed in Table 2. The latter indicates that ballistic transport at those frequencies is dominated by the matrix and that the inclusions have little effect despite the distortion of the wave front. However, it is worth mentioning that a previous study observed a decrease of MFP at low frequencies for a similar system with smaller spherical inclusions and the same crystalline volume fraction [15]. This difference might be explained by the increased density of scatterers that amplifies the interfacial effects or by specific coherent effects as the wavelength is close to the size of the spheres in this case. To conclude on this point, the most effective way to decrease the transmission of low frequency WP relative to bulk a-Si in these nanocomposites is to introduce pores.

While only a few differences appear between the configurations at low frequencies, at high frequencies, strong disparities between the nanocomposites become clear. At high frequencies, the MFP in a-Si is small [43], and there is an impedance mismatch between a-Si and c-Si for both polarizations (see the middle panel of Figure 3). As a result, the WP is strongly attenuated in the matrix but travels well through the structural percolation at high frequencies. A previous study has shown a similar behavior for NWs with an amorphous shell [67].

Interestingly, if the MFP is affected by the shape of the NIs, the diffusivity is not. All the configurations that include NIs have a very similar diffusivity. This diffusivity is distinctively higher than the bulk a-Si one (see Figure 4). A diffusivity increase caused by the addition of NIs has already been observed [15]. The only strategy to decrease the diffusivity of a-Si seems to be the creation of pores.

To summarize the ballistic transport properties, NIs were already known to affect the transmission of phonons, for instance, small spherical NIs act as a low pass filter [4], and here we show that if there is a structural percolation in the nanocomposite it can be used as a bandpass filter centered at 10 THz.

### 4.3. Validity of the Hypothesis Made

Equations (3) and (4) rely on different hypotheses. In this section, the validity of these hypotheses along with the possible origins of the discrepancies between the models is reviewed.

First, both the diffusive and the propagative contributions are considered at all frequencies. In previous works, the different contributions were separated either based on frequency ranges or on the periodicity of the modes [10]. Here, both contributions are included for all the frequencies considered. This is motivated by the fact that both a propagative and a diffusive part appear at all the observed frequencies for our configurations (see Table 2 and Table 3). This contributes to the overestimation of the thermal conductivity by the kinetic theory. Indeed, some modes are considered twice, once as diffusive and once as propagative. This is especially true in the low-frequency range where both MFP and diffusivity are high. In such a regime, the relative contributions of expressions (3) and (4) should be weighted.

Secondly, the propagative contribution is also very likely overestimated. This overestimation already appears for the bulk a-Si for which the propagons are expected to contribute up to 40% of κ [9] and our model gives 50%. This overestimation can be attributed to the lack of a cut-off frequency for the propagative contribution as previously discussed. The effect is much more marked for the STC and NW-M nanocomposites; for those, only a small fraction of the system (restricted to the center of the crystalline part) takes part to the ballistic transport at high frequencies (see Table 3). The transport only happens in the structural percolation and not in the whole nanocomposite. A manifestation of this phenomenon also appears in Figure 1; part of the energy is scattered and part of it travels ballistically. The diffusive behavior is visible through the gradual flattening of the central peak (0–10 nm). The propagative behavior is given by the lobe shifting through the sample. This lobe corresponds to the WP travelling in the structural percolation. However, in Equation (3), it is assumed that the whole configuration contributes to $\kappa_{P}$. This leads to an overestimation of $\kappa_{P}$, especially at high frequencies where non-propagating modes are taken into account in the VDOS but do not contribute to the ballistic transport.

A confinement effect, inducing the decrease of the group velocity, has been predicted for free NWs [68] and observed experimentally recently [69]. Such effects are not visible in our case (see Figure 3). However, as the group velocity is extracted from the fitting of the dispersion relation by a sine function, the eventual confinement effects impacting the low frequencies may be neglected due to the low wave-vector resolution.

Moreover, in the WP simulations, the propagation direction is aligned with the structural percolation. This alignment decreases the interactions of the WP with the interface and with the branches of the NW-M and STC perpendicular to the propagation at the crossings. This may artificially increase the MFP measured by WP propagation. As the boundary scattering is known to be the main factor limiting the thermal conductivity of NWs, a model has been developed to take it into account by considering a specularity parameter for reflections at the interface [70]. It has even been shown recently for argon NWs that this specularity parameter has a stronger effect on the thermal conductivity than the confinement effect [71]. Moreover, due to the geometry, the impact of boundary scattering might be even more important, especially in the case of the NW-M, where back scattering at intersections is expected to play an important role [28], whereas no impact of the intersection of the NW is visible in Table 3.

For comparison purposes, the MFP can also be estimated through the DSF thanks to the damped harmonic oscillator model (see Appendix A). However, this method is known to give lower lifetime than the estimation through WP amplitude decay rate [43]. Additionally, the DSF is an averaged quantity computed over the whole unit cells represented in Table 1. Thus, it cannot take into account the longer MFP due to transport in the structural percolation. As a result, if the MFP computed through the DSF is used to estimate $\kappa_{P}$, the hierarchy of κ between the nanocomposites obtained with EMD is not reproduced.

Finally, the hypotheses made on the effect of temperature are important. Namely, the MFP and the diffusivity are computed at 0 K, and then for $\kappa_{P}$ a phonon–phonon lifetime term is added to take into account the thermal effects. This phonon–phonon scattering parameter is approximated thanks to empirical coefficients derived for bulk c-Si. These coefficients were already successfully used for NWs, albeit NWs with larger characteristic dimensions than in the present study [45]. Alternatively, one may consider the expression for Umklapp processes derived by Klemens [72]. Moreover, the phonon–phonon scattering in amorphous materials is negligible, its effect being small in front of the effect of disorder. Thus, the bulk c-Si scattering coefficients seem to be the best available. Nonetheless, these parameters might be impacted by the interfaces and size effects. Interfaces are known to increase electron–phonon coupling [73] and could also increase the phonon–phonon scattering. The diffusivity can also be influenced by the temperature. To avoid using temperature correction coefficients, the WP propagation simulations could be performed at higher temperature. However, at higher temperature, the amplitude of the impulsion has to be increased in order to distinguish the WP from the thermal agitation. This larger impulsion may induce other bias, such as the overestimation of the effect of anharmonicity.

All those factors lead to an overestimation of the thermal conductivity computed through the kinetic theory and particularly of the propagative part. In future work, the estimation of the thermal conductivity using the kinetic theory could be improved by including the effect of the reflection at the interface, for instance by introducing a specularity parameter [70].

## 5. Conclusions

The vibrational and thermal properties of gradually interconnected c-Si NIs in an a-Si host matrix were studied, with the goal of gaining a better understanding of the effects of a crystalline continuity at a constant NI volume fraction. WP simulations revealed that the structural percolation has a strong impact on the transmission of energy at high frequencies (8–12 THz), the MFP being increased by an order of magnitude in the case of structural percolation. The interconnection also results in a thermal conductivity increase. This enhancement appears for the two methods used in our paper for the estimation of κ: the WP method (kinetic theory) and EMD computation. However, the kinetic theory predicts a twofold increase of κ between the non-interconnected NIs and the interconnected NIs, while the EMD simulations predict a more modest increase of 20%. More generally, the use of Equations (3) and (4) seems to overestimate κ, especially its propagative part $\kappa_{P}$. This difference between the predictions of the two methods has multiple roots: the contribution of all frequencies to both $\kappa_{D}$ and $\kappa_{P}$, the overestimation of the MFP due to alignment effects and the incomplete consideration of temperature effects. This leads us to conclude that, if ballistic transport can be observed at high frequencies for percolating NIs, it does not induce a marked absolute thermal conductivity increase. This kind of configurations could thus be used for applications where a low κ is needed while keeping the coherent transport of phonons at high frequencies. Such properties could be useful for information processing or phonons focusing in a structure.

## Figures and Tables

**Table 1:** Freestanding nanoparticles (first row) and a cross-section of NIs embedded in an a-Si matrix (in dark gray) cross-section (second row). In each case, the NI represents 30% of the volume of simulation cell.

Pore	Sphere	SC	STC	NW-M
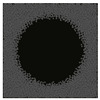	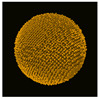	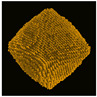	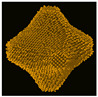	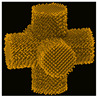
	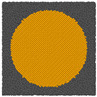	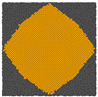	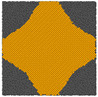	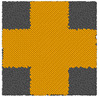

**Table 2:** Cross-sectional view of a WP going through the different systems after a longitudinal excitation at 2 THz (first part of the table) or a transverse excitation at the same frequency (second part of the table) every 0.9 ps. The first line represents the geometry of the cross-sections at the middle of simulation box with inclusions in yellow and matrix in dark grey. The color scale going from 0 (blue) to $3\times 10^{-9}$ eV (dark red) gives the atomic kinetic energy.

	a-Si	Pore	Sphere	SC	STC	NW-M
	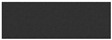	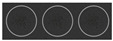	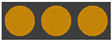	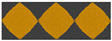	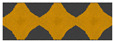	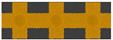
Time	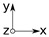	**Longitudinal**	2 THz	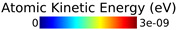		
0.6 ps	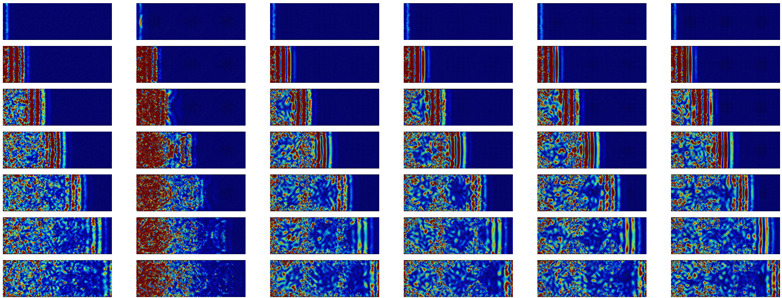					
1.5 ps						
2.4 ps						
3.3 ps						
4.2 ps						
5.1 ps						
6.0 ps						
Time	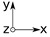	**Transverse**	2 THz	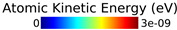		
0.6 ps	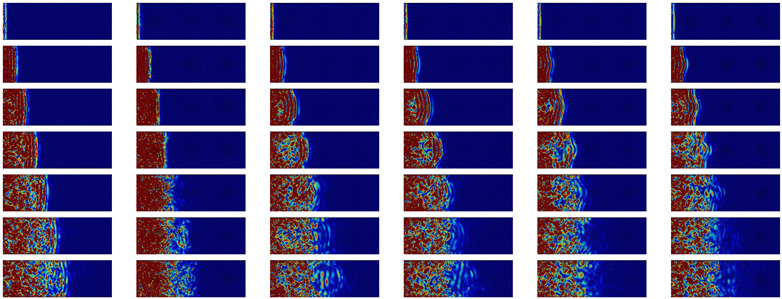					
1.5 ps						
2.4 ps						
3.3 ps						
4.2 ps						
5.1 ps						
6.0 ps						

**Table 3:** Cross-sectional view of a WP going through the different systems after a longitudinal excitation at 10 THz (first part of the table) or a transverse excitation at 4 THz (second part of the table) every 0.9 ps. The first line represents the geometry of the cross-sections at the middle of simulation box with inclusions in yellow and matrix in dark grey. The color scale going from 0 (blue) to $3\times 10^{-9}$ eV (dark red) gives the atomic kinetic energy.

	a-Si	Pore	Sphere	SC	STC	NW-M
	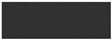	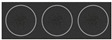	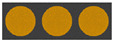	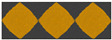	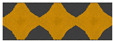	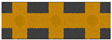
Time	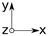	**Longitudinal**	10 THz	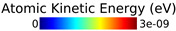		
0.6 ps	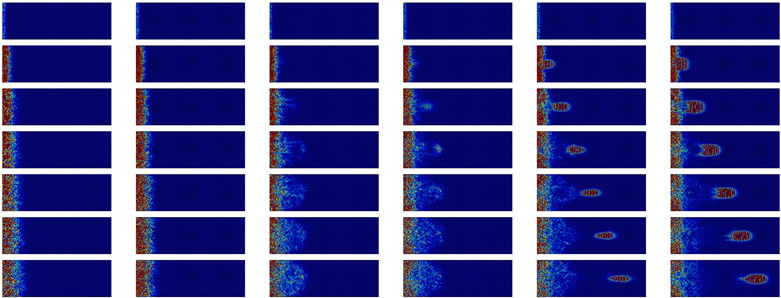					
1.5 ps						
2.4 ps						
3.3 ps						
4.2 ps						
5.1 ps						
6.0 ps						
Time	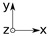	**Transverse**	4 THz	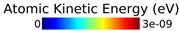		
0.6 ps	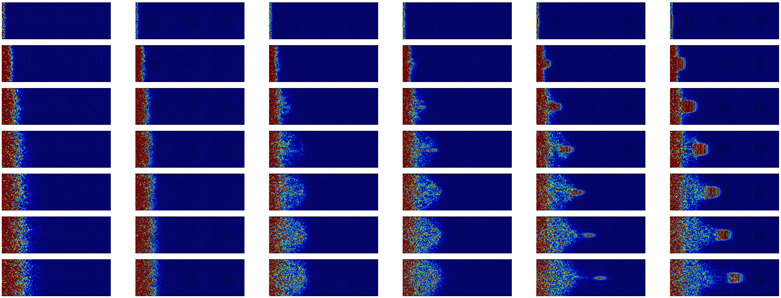					
1.5 ps						
2.4 ps						
3.3 ps						
4.2 ps						
5.1 ps						
6.0 ps						

**Table 4:** Thermal conductivity in $W\;m^{-1}K^{-1}$ at 300 K for the different configurations, computed with EMD or estimated with Equations (3) and (4).

	Am	Pore	S	SC	STC	NW-M
EMD	1.9 ± 0.2	0.89 ± 0.04	2.3 ± 0.1	2.3 ± 0.2	2.5 ± 0.2	2.7 ± 0.1
WP w $\tau_{ph-ph}$	2.5	1.3	4.0	4.1	7.2	8.9
WP w/o $\tau_{ph-ph}$	2.6	1.3	4.1	4.2	8.7	14.0

**Figure A1:**
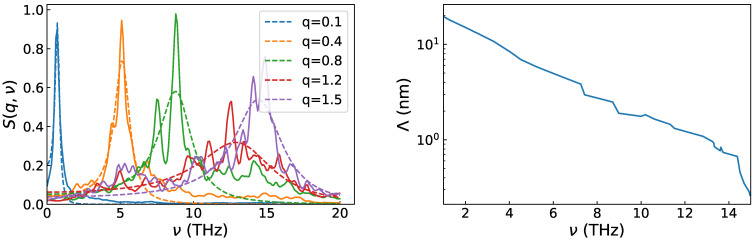
$S_{\eta }\left(q,\omega \right)$ as fitted from Equation (A1) (**left**) and resulting MFP (**right**) for the NW-M configuration for the longitudinal polarization.

**Figure A2:**
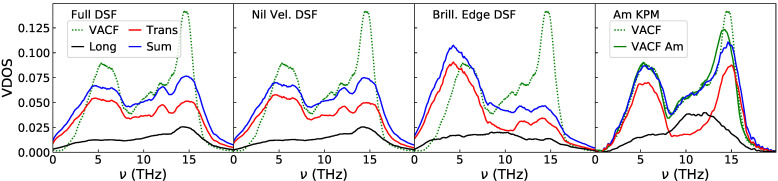
VDOS computed using different methods. The red and blue lines correspond, respectively, to the transverse and longitudinal polarizations. The solid blue and green dashed lines correspond to the total VDOS computed, respectively, via the DSF or the VACF. The first panel starting from the left uses the full DSF, the second the DSF limited to the theoretical wave-vector limit of the BZ of c-Si, the third the DSF limited to the wave vector giving a 0 velocity and the last the results using the KPM for an amorphous sample.

## Appendix A. Damped Harmonic Oscillator

The DSF can be used to extract both the dispersion relation and the lifetime of phonons. To this end, the low-frequency region of the DSF can be fitted with the damped harmonic oscillator (DHO) model [42]:

(A1) $$S_{\eta }\left(q,\omega \right)=\frac{A}{{\left(\omega^{2}-\omega_{\eta }^{2}\left(q\right)\right)}^{2}+\omega^{2}\Gamma_{\eta }^{2}}$$

with $\Gamma_{\eta }=1/\tau_{\eta }$ the inverse lifetime, $\omega_{\eta }\left(q\right)$ the phonon dispersion, *A* the amplitude and η labeling either the longitudinal or transverse polarization. The parameters of Equation (A1) are fitted to match the DSF obtained with Equation (12) for every wave vector. This fit is realized on the DSF convoluted with the experimental resolution [4]. Thus, this model enables the computation of both the lifetime and the dispersion relation using the DSF only.

The result of the fitting to a DHO model for a longitudinal polarization for the NW-M is displayed in Figure A1 (left) for a few wave vectors. The expression (A1) seems to fit to the DSF computed via Equation (12) reasonably well at low frequencies. However, as the frequency increases, the DSF is increasingly noisy, degrading the fit quality. The MFP can also be extracted (Figure A1, left). It then appears that the MFP peak at 10 THz observed with the WP disappears. The MFP computed with this method steadily decreases. This discrepancy can be attributed to the low-frequency limit of validity of this fit discussed in [42] or to the fact that this MFP is computed using a quantity averaged over the whole sample. This spatial average cannot capture the effect of the structural percolation showcased with the WP method.

The successive fits of different wave-vectors enable the computation of the dispersion relations $\omega_{\eta }\left(q\right)$, from which the group velocity can be computed. However, the method includes more parameters to fit than the method described in Section 2.6 and is thus a less robust approach.

## Appendix B. VDOS Estimated with DSF, VACF or KPM

The VDOS can be estimated thanks to the integral of the DSF over the wave vectors. This allows a distinction between the different wave-vector directions and polarizations. To consider the anisotropy of c-Si, the sum for the different vector directions of the Brillouin zone (BZ) is considered. The VDOS obtained are then filtered using the Savitzky–Golay polynomial filter.

However, a choice has to be made regarding the range of wave-vectors considered for the integration. The different possibilities tested are displayed in Figure A2. They are compared with the results of the VACF, which serve as a reference point.

The first possibility is to consider the whole DSF computed, that is from 0 to 2.25 ${Å\;}^{-1}$ (“Full DSF” in Figure A2). Another possibility is to consider the point at which the estimated group velocity of the phonons is nil (“Nil Vel. DSF” in Figure A2), relying on the fact that near the BZ limit the velocity is nil. The last possibility is to consider the theoretical end of the BZ computed for the lattice parameter used (“Brill. Edge DSF” in Figure A2). Another comparison point for the study is the separation of transverse and longitudinal VDOS by the KPM [49] on a fully amorphous sample, given that the configurations are mostly amorphous (70% of a-Si and 30% of c-Si).

When comparing the different VDOS, it appears that the total VDOS computed using DSF does not match with the VACF results, whatever wave vector limit is used. If all the peaks seem to be present, their relative size does not match, and they are flattened. This is even more marked when considering the theoretical end of the BZ (third panel in Figure A2). The failure to reproduce the VDOS using the DSF can partially be attributed to the fact that the BZ is not defined for the amorphous Si.

On the other hand, the VDOS of a-Si computed via the KPM matches comparatively well the VDOS of the NW-M configuration computed via VACF. However, in this case, some differences arise after 12 THz, but, given the reduced MFP at those frequencies, its impact on the computation of $\kappa_{P}$ is negligible.

## References

[1] Kim W., Zide J., Gossard A., Klenov D., Stemmer S., Shakouri A., Majumdar A. (2006). Thermal Conductivity Reduction and Thermoelectric Figure of Merit Increase by Embedding Nanoparticles in Crystalline Semiconductors. Phys. Rev. Lett..

[2] Zhu T.J., Yan F., Zhao X.B., Zhang S.N., Chen Y., Yang S.H. (2007). Preparation and thermoelectric properties of bulkin situnanocomposites with amorphous/nanocrystal hybrid structure. J. Phys. D Appl. Phys..

[3] Murray D.B., Saviot L. (2004). Phonons in an inhomogeneous continuum: Vibrations of an embedded nanoparticle. Phys. Rev. B.

[4] Damart T., Giordano V.M., Tanguy A. (2015). Nanocrystalline inclusions as a low-pass filter for thermal transport in *a*-Si. Phys. Rev. B.

[5] Allen P.B., Feldman J.L. (1993). Thermal conductivity of disordered harmonic solids. Phys. Rev. B.

[6] Allen P.B., Feldman J.L., Fabian J., Wooten F. (1999). Diffusons, locons and propagons: Character of atomie yibrations in amorphous Si. Philos. Mag. B.

[7] Seyf H.R., Henry A. (2016). A method for distinguishing between propagons, diffusions, and locons. J. Appl. Phys..

[8] Beltukov Y.M., Kozub V.I., Parshin D.A. (2013). Ioffe-Regel criterion and diffusion of vibrations in random lattices. Phys. Rev. B.

[9] Larkin J.M., McGaughey A.J.H. (2014). Thermal conductivity accumulation in amorphous silica and amorphous silicon. Phys. Rev. B.

[10] DeAngelis F., Muraleedharan M.G., Moon J., Seyf H.R., Minnich A.J., McGaughey A.J.H., Henry A. (2019). Thermal Transport in Disordered Materials. Nanoscale Microscale Thermophys. Eng..

[11] Sääskilahti K., Oksanen J., Tulkki J., McGaughey A.J.H., Volz S. (2016). Vibrational mean free paths and thermal conductivity of amorphous silicon from non-equilibrium molecular dynamics simulations. AIP Adv..

[12] Luo H., Gravouil A., Giordano V., Tanguy A. (2019). Thermal Transport in a 2D Nanophononic Solid: Role of bi-Phasic Materials Properties on Acoustic Attenuation and Thermal Diffusivity. Nanomaterials.

[13] Termentzidis K., Giordano V.M., Katsikini M., Paloura E., Pernot G., Verdier M., Lacroix D., Karakostas I., Kioseoglou J. (2018). Enhanced thermal conductivity in percolating nanocomposites: A molecular dynamics investigation. Nanoscale.

[14] Zhang H., Minnich A.J. (2015). The best nanoparticle size distribution for minimum thermal conductivity. Sci. Rep..

[15] Tlili A., Giordano V.M., Beltukov Y.M., Desmarchelier P., Merabia S., Tanguy A. (2019). Enhancement and anticipation of the Ioffe–Regel crossover in amorphous/nanocrystalline composites. Nanoscale.

[16] Wang M., Pan N. (2008). Predictions of effective physical properties of complex multiphase materials. Mater. Sci. Eng. R Rep..

[17] Fang K.C., Weng C.I., Ju S.P. (2006). An investigation into the structural features and thermal conductivity of silicon nanoparticles using molecular dynamics simulations. Nanotechnology.

[18] Nan C.W., Birringer R., Clarke D.R., Gleiter H. (1997). Effective thermal conductivity of particulate composites with interfacial thermal resistance. J. Appl. Phys..

[19] Hofmeister H., Tan G., Dubiel M. (2005). Shape and internal structure of silver nanoparticles embedded in glass. J. Mater. Res..

[20] Vasudevan S., Fullerton-Shirey S.K. (2019). Effect of Nanoparticle Shape on the Electrical and Thermal Properties of Solid Polymer Electrolytes. J. Phys. Chem. C.

[21] Jabbari F., Rajabpour A., Saedodin S. (2017). Thermal conductivity and viscosity of nanofluids: A review of recent molecular dynamics studies. Chem. Eng. Sci..

[22] Miura A., Zhou S., Nozaki T., Shiomi J. (2015). Crystalline—Amorphous Silicon Nanocomposites with Reduced Thermal Conductivity for Bulk Thermoelectrics. ACS Appl. Mater. Interfaces.

[23] Nakamura Y., Isogawa M., Ueda T., Yamasaka S., Matsui H., Kikkawa J., Ikeuchi S., Oyake T., Hori T., Shiomi J. (2015). Anomalous reduction of thermal conductivity in coherent nanocrystal architecture for silicon thermoelectric material. Nano Energy.

[24] Nakamura Y. (2018). Nanostructure design for drastic reduction of thermal conductivity while preserving high electrical conductivity. Sci. Technol. Adv. Mater..

[25] Huang Y., Hu J., Yao Y., Zeng X., Sun J., Pan G., Sun R., Xu J.B., Wong C.P. (2017). Manipulating Orientation of Silicon Carbide Nanowire in Polymer Composites to Achieve High Thermal Conductivity. Adv. Mater. Interfaces.

[26] Car D., Wang J., Verheijen M.A., Bakkers E.P.A.M., Plissard S.R. (2014). Rationally Designed Single-Crystalline Nanowire Networks. Adv. Mater..

[27] Ma D., Ding H., Meng H., Feng L., Wu Y., Shiomi J., Yang N. (2016). Nano-cross-junction effect on phonon transport in silicon nanowire cages. Phys. Rev. B.

[28] Verdier M., Lacroix D., Termentzidis K. (2018). Thermal transport in two- and three-dimensional nanowire networks. Phys. Rev. B.

[29] Kimmer C., Aubry S., Skye A., Schelling P.K. (2007). Scattering of phonons from a high-energy grain boundary in silicon: Dependence on angle of incidence. Phys. Rev. B.

[30] Yang L., Minnich A.J. (2017). Thermal transport in nanocrystalline Si and SiGe by ab initio based Monte Carlo simulation. Sci. Rep..

[31] Hori T., Shiomi J., Dames C. (2015). Effective phonon mean free path in polycrystalline nanostructures. Appl. Phys. Lett..

[32] Stukowski A. (2009). Visualization and analysis of atomistic simulation data with OVITO—The Open Visualization Tool. Model. Simul. Mater. Sci. Eng..

[33] Maras E., Trushin O., Stukowski A., Ala-Nissila T., Jónsson H. (2016). Global transition path search for dislocation formation in Ge on Si(001). Comput. Phys. Commun..

[34] Plimpton S. (1995). Fast Parallel Algorithms for Short-Range Molecular Dynamics. J. Comput. Phys..

[35] Vink R., Barkema G., van der Weg W., Mousseau N. (2001). Fitting the Stillinger—Weber potential to amorphous silicon. J. Non-Cryst. Solids.

[36] France-Lanord A., Merabia S., Albaret T., Lacroix D., Termentzidis K. (2014). Thermal properties of amorphous/crystalline silicon superlattices. J. Phys. Condens. Matter.

[37] Kubo R. (1957). Statistical-mechanical theory of irreversible processes. I. General theory and simple applications to magnetic and conduction problems. J. Phys. Soc. Jpn..

[38] Surblys D., Matsubara H., Kikugawa G., Ohara T. (2019). Application of atomic stress to compute heat flux via molecular dynamics for systems with many-body interactions. Phys. Rev. E.

[39] Schelling P.K., Phillpot S.R., Keblinski P. (2002). Phonon wave-packet dynamics at semiconductor interfaces by molecular-dynamics simulation. Appl. Phys. Lett..

[40] Ashcroft N.W., Mermin N.D. (1976). Solid State Physics.

[41] Larkin J.M., McGaughey A.J.H. (2013). Predicting alloy vibrational mode properties using lattice dynamics calculations, molecular dynamics simulations, and the virtual crystal approximation. J. Appl. Phys..

[42] Beltukov Y.M., Fusco C., Parshin D.A., Tanguy A. (2016). Boson peak and Ioffe-Regel criterion in amorphous siliconlike materials: The effect of bond directionality. Phys. Rev. E.

[43] Beltukov Y.M., Parshin D.A., Giordano V.M., Tanguy A. (2018). Propagative and diffusive regimes of acoustic damping in bulk amorphous material. Phys. Rev. E.

[44] Callaway J. (1959). Model for Lattice Thermal Conductivity at Low Temperatures. Phys. Rev..

[45] Yang F., Dames C. (2013). Mean free path spectra as a tool to understand thermal conductivity in bulk and nanostructures. Phys. Rev. B.

[46] Boon J.P., Yip S. (1991). Molecular Hydrodynamics.

[47] Dove M.T. (1993). Time correlation functions. Introduction to Lattice Dynamics.

[48] Savitzky A., Golay M.J.E. (1964). Smoothing and Differentiation of Data by Simplified Least Squares Procedures. Anal. Chem..

[49] Beltukov Y.M., Fusco C., Tanguy A., Parshin D.A. (2015). Transverse and longitudinal vibrations in amorphous silicon. J. Phys. Conf. Ser..

[50] Weiße A., Wellein G., Alvermann A., Fehske H. (2006). The kernel polynomial method. Rev. Mod. Phys..

[51] Rycroft C.H. (2007). Multiscale Modeling in Granular Flow. Ph.D. Thesis.

[52] Chantrenne P., Termentzidis K. (2012). Prediction of the thermal conductivity of SiC nanowires with kinetic theory of gases. Phys. Status Solidi A.

[53] Li D., Wu Y., Kim P., Shi L., Yang P., Majumdar A. (2003). Thermal conductivity of individual silicon nanowires. Appl. Phys. Lett..

[54] Zeller R.C., Pohl R.O. (1971). Thermal Conductivity and Specific Heat of Noncrystalline Solids. Phys. Rev. B.

[55] Cahill D.G., Pohl R.O. (1988). Lattice Vibrations and Heat Transport in Crystals and Glasses. Annu. Rev. Phys. Chem..

[56] Tretiakov K.V., Scandolo S. (2004). Thermal conductivity of solid argon from molecular dynamics simulations. J. Chem. Phys..

[57] Schelling P.K., Phillpot S.R., Keblinski P. (2002). Comparison of atomic-level simulation methods for computing thermal conductivity. Phys. Rev. B.

[58] Lv W., Henry A. (2016). Direct calculation of modal contributions to thermal conductivity via Green–Kubo modal analysis. New J. Phys..

[59] Minnich A., Chen G. (2007). Modified effective medium formulation for the thermal conductivity of nanocomposites. Appl. Phys. Lett..

[60] Termentzidis K., Isaiev M., Salnikova A., Belabbas I., Lacroix D., Kioseoglou J. (2018). Impact of screw and edge dislocations on the thermal conductivity of individual nanowires and bulk GaN: A molecular dynamics study. Phys. Chem. Chem. Phys..

[61] Cahill D.G., Katiyar M., Abelson J.R. (1994). Thermal conductivity of a-Si:H thin films. Phys. Rev. B.

[62] Isaiev M., Newby P.J., Canut B., Tytarenko A., Lishchuk P., Andrusenko D., Gomès S., Bluet J.M., Fréchette L.G., Lysenko V. (2014). Thermal conductivity of partially amorphous porous silicon by photoacoustic technique. Mater. Lett..

[63] Massoud A.M., Chapuis P.O., Canut B., Bluet J.M. (2020). Thermal conductivity of irradiated porous silicon down to the oxide limit investigated by Raman thermometry and scanning thermal microscopy. J. Appl. Phys..

[64] Hamilton R.L., Crosser O.K. (1962). Thermal Conductivity of Heterogeneous Two-Component Systems. Ind. Eng. Chem. Fundam..

[65] Yang L., Zhang Q., Cui Z., Gerboth M., Zhao Y., Xu T.T., Walker D.G., Li D. (2017). Ballistic Phonon Penetration Depth in Amorphous Silicon Dioxide. Nano Lett..

[66] Yang L., Latour B., Minnich A.J. (2018). Phonon transmission at crystalline-amorphous interfaces studied using mode-resolved atomistic Green’s functions. Phys. Rev. B.

[67] Desmarchelier P., Tanguy A., Termentzidis K. (2021). Thermal rectification in asymmetric two-phase nanowires. Phys. Rev. B.

[68] Zou J., Balandin A. (2001). Phonon heat conduction in a semiconductor nanowire. J. Appl. Phys..

[69] Kargar F., Debnath B., Kakko J.P., Säynätjoki A., Lipsanen H., Nika D.L., Lake R.K., Balandin A.A. (2016). Direct observation of confined acoustic phonon polarization branches in free-standing semiconductor nanowires. Nat. Commun..

[70] Volz S.G., Chen G. (1999). Molecular dynamics simulation of thermal conductivity of silicon nanowires. Appl. Phys. Lett..

[71] Tretiakov K.V., Hyżorek K. (2021). Role of the phonon confinement effect and boundary scattering in reducing the thermal conductivity of argon nanowire. J. Chem. Phys..

[72] Klemens P. (1958). Thermal Conductivity and Lattice Vibrational Modes.

[73] Luh D.A., Miller T., Paggel J.J., Chiang T.C. (2002). Large Electron-Phonon Coupling at an Interface. Phys. Rev. Lett..

